# Prognostic Potential of Liver Enzymes in Patients With COVID-19 at the Leishenshan Hospital in Wuhan

**DOI:** 10.3389/fcimb.2021.636999

**Published:** 2021-11-16

**Authors:** Zeming Liu, Di Hu, Jinpeng Li, Qing Xia, Yan Gong, Zhengwei Li, Qian Wu, Meilin Yi, Yihui Huang, Meng Wu, Liang Guo, Xiaohui Wu

**Affiliations:** ^1^ Department of Plastic and Cosmetic Surgery, Tongji Hospital, Tongji Medical College, Huazhong University of Science and Technology, Wuhan, China; ^2^ Department of Plastic Surgery, Zhongnan Hospital of Wuhan University, Wuhan, China; ^3^ Department of Thyroid and Breast Surgery, Zhongnan Hospital of Wuhan University, Wuhan, China; ^4^ Department of Respiratory Medicine, Shanghai 10th People’s Hospital, Tongji University, Shanghai, China; ^5^ Department of Biological Repositories, Zhongnan Hospital of Wuhan University, Wuhan, China; ^6^ Department of Neurosurgery, Zhongnan Hospital of Wuhan University, Wuhan, China; ^7^ Department of Burn and Plastic Surgery, College of Traditional Chinese Medicine, Three Gorges University & Yichang Hospital of Traditional Chinese Medicine, Yichang, China; ^8^ Department of Ultrasound, Zhongnan Hospital of Wuhan University, Wuhan, China

**Keywords:** AST - aspartate transaminase, ALT - alanine transaminase, AST/ALT, aspartate aminotransferase/alanine aminotransferase, COVID-19, pneumonia

## Abstract

**Background:**

Coronavirus disease 2019 (COVID-19) has evolved into a pandemic. We hypothesized that biochemical indicators of liver function may help determine the prognosis of COVID-19 patients.

**Methods:**

Patient information was collected from the Wuhan-Leishenshan hospital. Logistic and Cox regression analyses, Kaplan-Meier curves, and Curve fitting were used to determine the correlation between elevated levels of aspartate transaminase (AST), alanine transaminase (ALT), and AST/ALT and severity of disease/mortality.

**Results:**

Logistic and Cox regression analyses and Kaplan-Meier survival curves showed that COVID-19 progression correlated with elevated levels of AST and AST/ALT. The odds ratios for elevated levels of AST and AST/ALT in patients were 0.818 (95% confidence interval [CI]: 0.274-2.441, P = 0.035) and 2.055 (95% CI: 1.269-3.327, P = 0.003), respectively; the hazard ratios were 4.195 (95% CI: 1.219-14.422, P = 0.023) and 3.348 (95% CI: 1.57-7.139, P = 0.002), respectively. The Kaplan-Meier survival curves demonstrated that patients with elevated AST and AST/ALT levels had a higher risk of developing severe COVID-19.

**Conclusion:**

Elevated AST and AST/ALT levels correlated with severity of COVID-19 and mortality. Liver function tests may help clinicians in determining the prognosis of patients undergoing treatment for COVID-19.

## Background

Since its first reported occurrence in Wuhan, coronavirus disease 2019 (COVID-19) has become an alarming cause of morbidity and mortality worldwide. This can be attributed to the median incubation time, ranging from 4 to 7 days, between SARS-CoV-2 infection and the appearance of symptoms. This delays timely treatment and allows the transmission of viral particles across the population (Huang et al., 2020; Khan et al., 2020; Vardhana and Wolchok, 2020). More than five million patients have received COVID-19 diagnose, among which >355,000 deaths have been attributed to the disease (Worldometer COVID-19 Data).

The lack of targeted therapy, and factors affecting disease progression and prognosis of COVID-19 have also complicated the outcome of patients (Hui et al., 2020; Pan et al., 2020). Previous studies have shown that patient age, comorbidities, lymphocytopenia, elevated levels of D-dimer, creatine kinase, high-sensitivity cardiac troponin I, and higher prothrombin time correlate with disease severity in patients with COVID-19 in the intensive care unit. Liver function tests have recently been shown to be an important diagnostic tool for COVID-19. The liver function tests of patients show abnormalities; however, a study has refuted the relevance of impaired liver function in patients with COVID-19 and states that liver activity may not have serious clinical consequences (Zhang et al., 2020). Conversely, there are also researchers have different opinions. About 14-53% of patients with COVID-19 were reported to hepatic dysfunction, particularly in those with severe disease (Jothimani et al., 2020). Considering these contradictory findings, it is imperative to determine the effect of liver function in the pathogenesis of COVID-19. In this study, we aimed to examine the correlation between prognosis of patients with COVID-19 and aspartate transaminase (AST), alanine transaminase (ALT), and AST/ALT levels. This will help elucidate the role of impaired liver function in the progression of COVID-19 and devise novel therapeutic strategies for clinical intervention.

## Methods

### Study Design and Participants

This retrospective study comprised 1,788 patients who were clinically diagnosed with COVID-19 between February 8 and March 19, 2020 at the Leishenshan Hospital in Wuhan. Their demographic characteristics, medical history and treatment, laboratory test results, and imaging data were obtained from the original patient medical records. Two physicians independently reviewed this data. This study was approved by the Research Ethics Commission of the Zhongnan Hospital, Wuhan University (approval number: 2020074), and patient consent was waived by the ethics committee as COVID-19 is a rapidly evolving infectious disease.

### Primary Outcomes

We used patient survival, severity of COVID-19 during patient hospitalization, and computed tomography (CT) images to determine the primary outcomes of infected patients. Survival status was the most important indicator. According to the seventh interim guidance for diagnosis and treatment of COVID-19 published by the Chinese National Health Commission (National Health Commission of the People’s Republic of China), COVID-19 severity was divided into four degrees: mild, common, severe, and critical. As there was only one patient with mild COVID-19, we combined the first two groups to form the mild/common group.

The chest CT images were examined and scored independently by two experienced radiologists using common guidelines based on previous studies and symptoms of COVID-19. A score of 1 included characteristic like ground-glass opacities, reticulations or cord-like changes, consolidations, and pleural effusions. Each feature was assigned one point, comprising score 1. Score 2 was generated based on the area of lung lobe involvement, from 0 to 4: no involvement, 0; <25% involvement, 1; 26%-50% involvement, 2; 51%-75% involvement, 3; and 76%-100% involvement, 4. The total score was the sum of scores 1 and 2.

### Statistical Analysis

Continuous variables that exhibited normal distribution were presented as mean ± standard deviation (SD) or interquartile range (IQR). Categorical variables were described as frequency rates and percentages. Differences between continuous variables were analyzed using independent *t*-tests or Mann-Whitney tests in patients with normal and elevated levels of ALT and AST. Chi-square tests were used to analyze differences between categorical variables in patients with normal and elevated levels of ALT and AST. Fisher’s exact test were used for expected counts of less than 5 patients.

We used univariate logistic and Cox regression analyses to determine whether elevated levels of ALT, AST, and AST/ALT influenced the prognosis of patients with COVID-19. During logistic regression analysis, we divided all patients into two groups based on disease severity. Group 1 included patients who were diagnosed with mild/common or severe disease, and group 2 comprised patients who were critically ill. Subsequently, we adjusted the logistic and Cox regression analyses for age, history of cardiovascular disease, and white blood cell, platelet, and lymphocyte counts.

We used Kaplan-Meier survival analyses with log-rank tests to understand patient survival. We used curve fitting analysis for patients with normal or elevated levels of AST and ALT to examine the changes in chest cavities for CT scores 1, and 2, and for total score, in a time-dependent manner. All statistical analyses were performed using SPSS, version 22.0 (IBM Corp., Armonk, NY, USA). Two-tailed P values < 0.05 were considered statistically significant.

## Results

### Demographics, Clinical Information, and Laboratory Testing

**Table 1** lists the demographic characteristics and symptoms of 1,788 patients with COVID-19. The numbers of male and female patients were approximately the same. The median and interquartile range for patient age were 59 and 49-68 years, respectively. Patients with normal and elevated levels of AST were of similar age. The median age of patients with normal levels of ALT was higher than that of patients with elevated ALT.

**Table 1:** Demographic characteristics and symptoms of the 1,788 patients with COVID-19.

Covariates	All patients, n (%)	ALT			AST		
		Normal, n (%)	Elevated, n (%)	P-value	Normal, n (%)	Elevated, n (%)	P-value
Gender				<0.001			0.056
Female	913 (52.50)	833 (91.24)	80 (8.76)		848 (92.88)	65 (7.12)	
Male	826 (47.50)	657 (79.54)	169 (20.46)		749 (90.68)	77 (9.32)	
Age, median (IQR)	59 (49-68)	60 (49-69)	54 (43-62)	<0.001	59 (49-68)	59 (47-69)	<0.001
Any comorbidity							
Cardiovascular disease	356 (19.91)	317 (89.04)	39 (10.96)	0.040	320 (89.89)	36 (10.11)	0.241
Pulmonary disease	89 (5.12)	79 (88.76)	10 (11.24)	0.394	82 (92.13)	7 (7.87)	0.915
Endocrine disease	137 (7.66)	124 (90.51)	13 (9.49)	0.090	132 (96.35)	5 (3.65)	0.033
Malignancy	64 (3.58)	54 (84.38)	10 (15.63)	0.771	56 (87.50)	8 (12.50)	0.251
Digest system disease	45 (2.52)	37 (82.22)	8 (17.78)	0.510	36 (80.00)	9 (20.00)	0.005
Neurological disease	56 (3.13)	51 (91.07)	5 (8.93)	0.238	49 (87.50)	7 (12.50)	0.284
Initial symptoms							
Fever or fatigue	620 (34.68)	516 (83.23)	104 (16.77)	0.035	547 (88.23)	73 (11.77)	<0.001
Respiratory symptoms	635 (35.51)	543 (85.51)	92 (14.49)	0.918	568 (89.45)	67 (10.55)	0.025
Digestive symptoms	82 (4.59)	70 (85.37)	12 (14.63)	0.945	72 (87.80)	10 (12.20)	0.228
Neurological symptoms	26 (1.45)	21 (80.77)	5 (19.23)	0.315	23 (88.46)	3 (11.54)	0.483
Other	23 (1.45)	23 (88.46)	3 (11.54)	0.474	26 (100)	0 (0)	0.161

There was no significant difference between comorbidities (**Table 1**), such as pulmonary disease, endocrine disease, malignancy, digestive system disease, and neurological disorders, in patients with normal or elevated levels of ALT. However, differential AST function in patients correlated with the presence of endocrine disease and abnormalities of the digestive system. Fever or fatigue was a differentiating factor between patients with normal and elevated levels of ALT and AST (**Table 1**). Furthermore, among the remaining physiological anomalies analyzed, symptoms of respiratory failure influenced disease progression in patients with normal and elevated levels of AST (**Table 1**).

**Tables 2**, **3** shown the correlation between biochemical laboratory results in patients with normal and elevated levels of ALT and AST. The majority of the parameters showed significant differences between patient groups. Moreover, treatment with antiviral drugs, corticosteroids, and traditional Chinese medicines affected patients with normal and elevated levels of ALT (**Table 4**). Anticoagulants, corticosteroids, vitamin C, and oxygen support (positive pressure nasal cannula, high-flow nasal cannula, and invasive mechanical ventilation) showed significant differences in patients with normal and elevated levels of AST (**Table 4**). Compared to differential levels of ALT, those of AST in patients correlated with various parameters.

**Table 2:** Laboratory test results of the patients with COVID-19.

Covariate	All patientsMedian (IQR)/n (%)	ALT				AST		
		Normal Median (IQR)/n (%)	Elevated Median (IQR)/n (%)		p-value	Normal Median (IQR)/n (%)	Elevated Median (IQR)/n (%)	p-value
Leucocyte count, × 10⁹/L	5.69 (4.70-6.90)	5.63 (4.64-6.84)	6.03 (5.03-7.32)		0.009	5.68 (4.70-6.87)	5.70 (4.81-7.11)	0.084
3.5-9.5	1592 (89.14)	1362 (85.55)	230 (14.45)			1463 (91.90)	129 (8.10)	
< 3.5	104 (5.82)	97 (93.27)	7 (6.370			93 (89.42)	11 (10.58)	
> 9.5	90 (5.04)	70 (77.78)	20 (22.22)			77 (85.56)	13 (14.44)	
Neutrophil count, × 10⁹/L	3.27 (2.53-4.25)	3.24 (2.50-4.23)	3.52 (2.77-4.52)		0.001	3.25 (2.52-6.87)	3.36 (2.61-4.54)	0.012
1.8-6.3	1558 (87.23)	1336 (85.75)	222 (14.25)			1433 (91.98)	125 (8.02)	
< 1.8	117 (6.55)	109 (93.16)	8 (6.84)			107 (91.45)	10 (8.55)	
> 6.3	111 (6.22)	84 (75.68)	27 (24.32)			93 (83.78)	18 (16.22)	
Lymphocyte count, × 10⁹/L	1.60 (1.24-1.99)	1.60 (.24-1.97)	1.64 (1.21-2.04)		0.130	1.62 (1.27-1.99)	1.47 (0.95-1.94)	<0.001
1.1-3.2	1462 (81.86)	1259 (86.11)	203 (13.89)			1361 (93.09)	101 (6.91)	
< 1.1	298 (16.69)	251 (84.23)	47 (15.77)			247 (82.89)	51 (17.11)	
> 3.2	26 (1.46)	19 (73.08)	7 (2.72)			25 (1.53)	1 (3.85)	
Erythrocyte count, × 10^12^/L	4.12 (3.77-4.49)	4.10 (3.73-4.44)	4.32 (3.29-4.75)		<0.001	4.12 (13.77-4.49)	4.12 (3.71-4.51)	0.499
4.3-5.8	639 (35.78)	512 (80.13)	127 (19.87)			583 (91.24)	56 (8.76)	
< 4.3	1136 (63.61)	1009 (65.99)	127 (11.18)			1041 (91.64)	95 (8.36)	
> 5.8	11 (0.62)	8 (72.73)	3 (27.27)			9 (81.82)	2 (18.18)	
Monocyte count, × 10⁹/L	0.50 (0.40-0.63)	0.50 (0.40-0.62)	0.55 (0.46-0.70)		<0.001	0.50 (0.40-0.63)	0.53 (0.41-0.70)	<0.001
0.1-0.6	1256 (70.32)	1102 (87.74)	154 (12.26)			1155 (91.96)	101 (8.04)	
< 0.1	6 (0.34)	5 (83.33)	1 (16.67)			6 (100.00)	0 (0)	
> 0.6	524 (29.34)	422 (80.53)	102 (19.47)			472 (90.08)	52 (9.92)	
Hemoglobin, g/L	126 (115-137)	124 (114-135)	133 (123-144)		<0.001	126 (115-137)	126 (116-140)	0.643
130.0-175.0	712 (39.87)	565 (79.35)	147 (20.65)			650 (91.29)	62 (8.71)	
< 130.0	1069 (59.85)	961 (89.90)	108 (10.10)			979 (91.58)	90 (8.42)	
> 175.0	5 (0.28)	3 (60.00)	2 (20.00)			4 (80.00)	1 (20.00)	
Platelet count, × 10^9^/L	229.00 (187.00-277.25)	228 (186-276)	234.00 (192.50-287.00)		0.121	229.00 (187.50-277.00)	230.00 (170.50-288.50)	<0.001
125.0-350.0	1556 (87.12)	1340 (87.64)	216 (13.88)			1434 (92.16)	122 (7.84)	
< 125.0	77 (4.31)	60 (77.92)	17 (22.08)			60 (77.92)	17 (22.08)	
> 350.0	153 (8.57)	129 (8.44)	24 (15.69)			139 (90.85)	14 (9.15)	
Albumin, g/L	37.70 (35.00-40.00)	37.60 (34.90-39.90)	38.30 (35.55-40.50)		0.109	37.80 (35.20-40.00)	36.90 (31.95-39.70)	0.380
40-55	450 (25.17)	375 (83.33)	75 (16.67)			416 (92.44)	34 (7.560	
<40	1338 (74.83)	1156 (75.51)	182 (13.60)			1219 (91.11)	119 (8.89)	
Total bilirubin, μmol/L	9.10 (7.00-12.00)	9.10 (6.90-11.90)	9.40 (7.20-12.95)		0.288	9.10 (7.00-11.90)	9.70 (6.85-13.90)	0.001
5.0-21.0	1595 (89.20)	1367 (85.69)	228 (14.31)			1470 (92.16)	125 (7.84)	
< 5.0	124 (6.94)	109 (87.90)	15 (12.10)			110 (88.71)	14 (11.29)	
> 21.0	69 (3.86)	55 (79.71)	14 (20.29)			55 (79.71)	14 (20.29)	
Creatinine, μmol/L	52.00 (36.00-75.00)	63.60 (54.00-76.00)	67.10 (57.70-78.10)		0.003	64.30 (54.70-76.50)	64.90 (52.30-72.45)	0.687
64.0-104.0	814 (45.52)	672 (82.53)	142 (17.47)			743 (91.28)	71 (8.72)	
< 64.0	879 (49.16)	774 (88.04)	105 (11.96)			807 (91.81)	72 (8.19)	
> 104.0	93 (5.22)	83 (89.25)	10 (10.75)			83 (89.25)	10 (10.75)	
Procalcitonin, ng/mL	0.04 (0.03-0.05)	0.04 (0.02-0.05)	0.05 (0.03-0.07)	<0.001		0.04 (0.03-0.05)	0.06 (0.04-0.11)	<0.001
<0.05	1000 (65.96)	902 (90.20)	98 (9.80)			952 (95.20)	48 (4.80)	
>=0.05	516 (34.04)	394 (76.36)	122 (23.64)			425 (82.36)	91 (17.64)	
Interleukin-6, pg/mL	1.50 (1.50-4.03)	1.50 (1.50-4.14)	1.5 (1.5-3.48)	0.695		1.50 (1.50-3.69)	2.72 (1.50-11.05)	<0.001
0-7.0	606 (83.59)	495 (83.33)	111 (84.73)			552 (91.09)	54 (8.91)	
>7.0	119 (16.41)	99 (83.19)	20 (16.81)			93 (78.15)	26 (21.85)	
SARS-CoV-19 IgM				0.031				0.560
NO	1569 (87.75)	1354 (86.30)	215 (13.70)			1437 (87.89)	132 (8.41)	
YES	219 (12.25)	177 (80.82)	42 (19.18)			198 (90.14)	21 (9.59)	
SARS-CoV-19 IgG				0.44				0.303
NO	1257 (70.30)	1090 (86.71)	167 (13.29)			1155 (81.89)	102 (8.11)	
YES	531 (29.70)	441 (83.05)	90 (16.95)			480 (90.40)	51 (9.60)	

**Table 3:** Blood coagulation in the patients with COVID-19.

Covariate	All patientsMedian (IQR)/n (%)	ALT			AST		
		Normal Median (IQR)/n (%)	Elevated Median (IQR)/n (%)	p-value	Normal Median (IQR)/n (%)	Elevated Median (IQR)/n (%)	p-value
Prothrombin time, s	11.30 (10.90-11.75)	11.30 (10.90-11.70)	11.30 (10.87-11.80)	0.788	11.30 (10.90-11.70)	11.45 (11.00-12.17)	<0.001
9.4-12.5	1469 (92.22)	1251 (85.16)	218 (14.84)		1353 (92.10)	116 (7.90)	
< 9.4	1 (0.06)	1 (100)	0 (0)		1 (100)	0 (0)	
> 12.5	123 (7.72)	107 (86.99)	16 (6.84)		99 (80.49)	24 (19.51)	
International Normalized Ratio	0.97 (0.93-1.01)	0.97 (0.93-1.01)	0.97 (0.93-1.02)	0.726	0.97 (0.93-1.01)	0.98 (0.94-1.05)	<0.001
0.8-1.3	1515 (95.10)	1294 (85.41)	221 (14.59)		1392 (91.88)	123 (8.12)	
< 0.8	19 (1.19)	15 (78.95)	4 (21.05)		19 (100)	0 (0)	
> 1.3	59 (3.70)	50 (84.75)	9 (15.25)		42 (91.19)	17 (28.81)	
Activated partial thromboplastin time, s	27.20 (24.60-30.40)	27.30 (24.70-30.50)	27.00 (24.27)	0.402	27.20 (24.60-30.30)	28.00 (25.12-31.92)	0.028
25.1-36.5	1044 (65.54)	889 (85.15)	155 (14.85)		949 (90.90)	95 (9.10)	
< 25.1	466 (29.25)	395 (84.76)	71 (15.24)		434 (93.13)	32 (6.87)	
> 36.5	83 (5.21)	75 (90.36)	8 (9.64)		70 (84.34)	13 (15.66)	
Fibrinogen, (g/L)	2.95 (2.51-3.73)	2.95 (2.51-3.68)	2.91 (2.51-3.87)	0.063	2.92 (2.51-3.68)	3.21 (2.54-4.08)	0.051
2.38-4.98	1183 (74.26)	1013 (85.63)	170 (14.37)		1079 (91.21)	104 (8.79)	
< 2.38	308 (19.34)	262 (85.06)	46 (14.94)		287 (93.18)	21 (6.82)	
> 4.98	102 (6.40)	84 (82.35)	18 (17.65)		87 (85.29)	15 (14.71)	
Thrombin time, s	17.60 (17.00-18.40)	17.60 (17.00-18.40)	17.70 (17.00-18.60)	0.079	17.70 (17.00-18.40)	17.60 (16.90-18.77)	0.165
<=16.6	243 (15.25)	206 (84.77)	37 (15.23)		216 (88.89)	27 (11.11)	
> 16.6	1350 (84.75)	1153 (85.41)	197 (14.59)		1237 (91.63)	113 (8.37)	
D-dimer, g/L	0.38 (0.21-0.90)	0.47 (0.23-1.51)	0.35 (0.20-0.89)		0.37 (0.21-0.87)	0.56 (0.23-1.65)	

**Table 4:** Treatment and outcome of the patients with COVID-19.

Covariate	All patients (%)	ALT			AST		
		Normal (%)	Elevated (%)	p-value	Normal (%)	Elevated (%)	p-value
Drugs							
Antibiotic	521 (29.14)	445 (85.41)	76 (14.59)	0.869	469 (90.02)	52 (9.98)	0.168
Antiviral drugs	869 (48.60)	758 (87.34)	110 (12.66)	0.044	803 (92.41)	66 (7.59)	0.157
Antimalarial drugs	139 (7.77)	116 (83.45)	23 (16.55)	0.447	124 (89.21)	15 (10.79)	0.327
Anticoagulants	131 (7.33)	112 (85.50)	19 (14.50)	0.965	108 (82.44)	23 (17.56)	<0.001
Corticosteroid	106 (5.93)	80 (75.47)	26 (24.53)	0.002	87 (82.08)	19 (17.92)	<0.001
Vitamin C	248 (13.87)	204 (82.26)	44 (17.74)	0.103	214 (86.29)	34 (13.71)	0.002
Traditional Chinese medicine	1533 (85.74)	1324 (86.73)	209 (13.63)	0.029	1409 (91.91)	124 (8.09)	0.083
Oxygen Support							
Low-flow nasal cannula	269 (15.04)	236 (87.73)	33 (12.27)	0.285	247 (91.82)	22 (8.18)	0.810
Positive pressure nasal cannula	34 (1.90)	25 (73.53)	9 (26.47)	0.042	26 (76.47)	8 (23.53)	0.002
High-flow nasal cannula	19 (1.06)	13 (68.42)	6 (31.58)	0.032	14 (73.68)	5 (26.32)	0.005
Invasive mechanical ventilation	6 (0.34)	3 (50)	3 (50)	0.013	4 (66.67)	2 (33.33)	0.030
ECMO	1	1 (100)	0 (0)	0.682	1 (100)	0 (0)	0.760
CT scores				0.618			0.028
1-4	75 (41.44)	63 (84.00)	12 (16.00)		68 (90.67)	7 (9.33)	
5-7	106 (58.56)	86 (81.13)	20 (18.87)		83 (78.30)	23 (21.70)	
Disease Progression				0.329			<0.001
Stableness/Hospitalization	15 (0.85)	12 (80.00)	3 (20.00)		12 (80.00)	3 (20.00)	
Improvement/Recover	1736 (98.30)	1488 (85.71)	248 (14.29)		1597 (91.99)	139 (8.01)	
Death	15 (0.85)	11 (73.33)	4 (26.67)		8 (53.33)	7 (46.67)	
Days in hospital, Median (IQR)	18 (13-24)						
ICU care	35 (1.96)	29 (82.86)	6 (17.14)	0.637	27 (77.14)	8 (22.86)	0.002
Severity on admission				0.715			0.005
Mild/Common	1478 (82.66)	1266 (85.66)	212 (14.34)		1362 (92.15)	116 (7.85)	
Severe	285 (15.94)	245 (85.96)	40 (14.04)		254 (89.12)	31 (10.88)	
Critical	25 (1.40)	20 (80)	5 (20)		19 (76)	6 (24)	
Severity at worst				0.807			<0.001
Mild/Common	931 (52.16)	802 (86.14)	129 (13.86)		874 (93.88)	57 (6.12)	
Severe	804 (45.04)	685 (85.20)	119 (14.80)		721 (89.68)	83 (10.32)	
Critical	50 (2.80)	42 (84.00)	8 (16.00)		37 (74.00)	13 (26.00)	

### Patient Prognosis

**Tables 5**, **6** shown the correlation between elevated levels of AST, ALT, and AST/ALT and disease severity/mortality in patients with COVID-19. Logistic and Cox regression analyses revealed that elevated levels of AST and AST/ALT, but not ALT, correlated with poor prognosis in patients with COVID-19 relative to the prognosis of patients with normal levels of AST and AST/ALT. After adjusting for age, history of cardiovascular disease, and white blood cell, platelet, and lymphocyte counts, the odds ratios for patients with COVID-19 with elevated levels of AST, ALT, and AST/ALT were 0.818 (95% confidence interval [CI]: 0.274-2.441, P = 0.035), 1.106 (95% CI: 0.777-1.575, P = 0.719), and 2.055 (95% CI: 1.269-3.327, P = 0.003), respectively. Hazard ratios for these patients were 4.195 (95% CI: 1.219-14.422, P = 0.023), 1.885 (95% CI: 0.450-7.904, P = 0.382), and 3.348 (95% CI: 1.57-7.139, P = 0.002), respectively.

**Table 5:** The odds ratio associated with elevated levels of AST, ALT, and AST/ALT and COVID-19 severity.

Variables	Univariate Analysis			Multivariate Analysis*		
	ORs	95% CI	p-value	ORs	95% CI	p-value
AST	4.003	2.079-7.707	<0.001	0.818	0.274-2.441	0.035
ALT	1.142	0.530-2.462	0.735	1.106	0.777-1.575	0.719
AST/ALT	2.967	1.947-4.523	<0.001	2.055	1.269-3.327	0.003

*Adjusted for age, history of cardiovascular disease, and white blood cell, platelet, and lymphocyte counts.

**Table 6:** The hazards ratio associated with elevated levels of AST, ALT, and AST/ALT and COVID-19 induced mortality.

Variables	Univariate Analysis			Multivariate Analysis*		
	HRs	95% CI	p-value	HRs	95% CI	p-value
AST	9.048	3.278-27.974	<0.001	4.193	1.219-14.422	0.023
ALT	2.114	0.683-6.733	0.192	1.886	0.450-7.904	0.382
AST/ALT	3.720	1.963-7.051	<0.001	3.348	1.570-7.139	0.002

*Adjusted for age, history of cardiovascular disease, and white blood cell, platelet, and lymphocyte counts.

**Figure 1:**
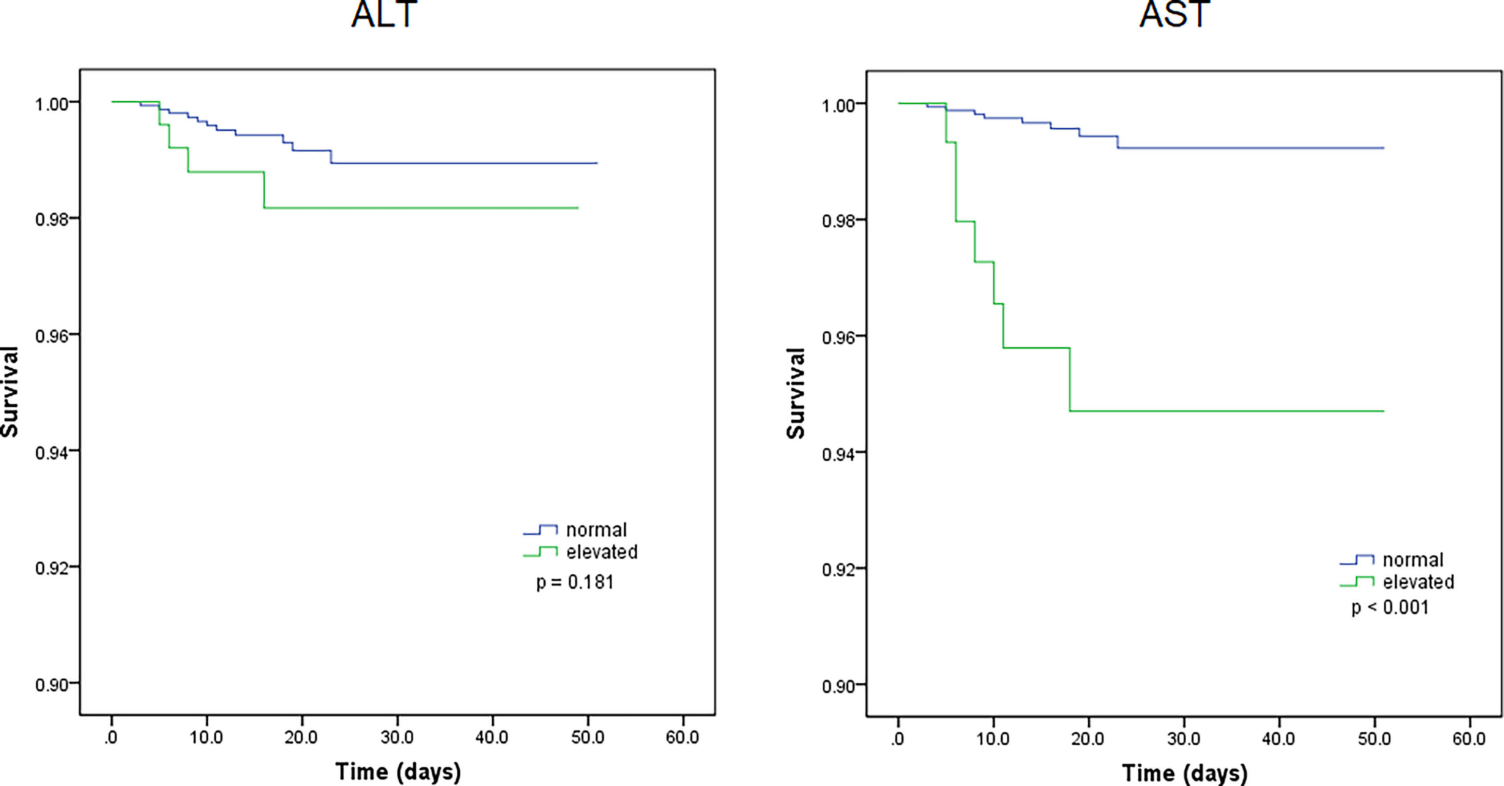
Kaplan-Meier survival curves for patients with normal and elevated levels of aspartate transaminase (AST) and alanine aminotransferase (ALT).

**Figure 2:**
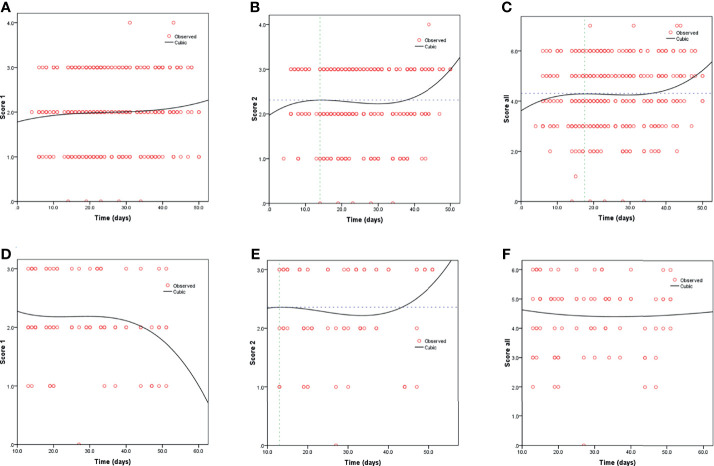
Fitting curves for COVID-19 patients with normal/elevated levels of AST based on CT score. Dynamic changes in patients with **(A)** CT score 1 and normal AST; **(B)** CT score 2 and normal AST; **(C)** total CT score and normal AST; **(D)** CT score 1 and elevated AST; **(E)** CT score 2 and elevated AST; and **(F)** total CT score and elevated AST.

**Figure 3:**
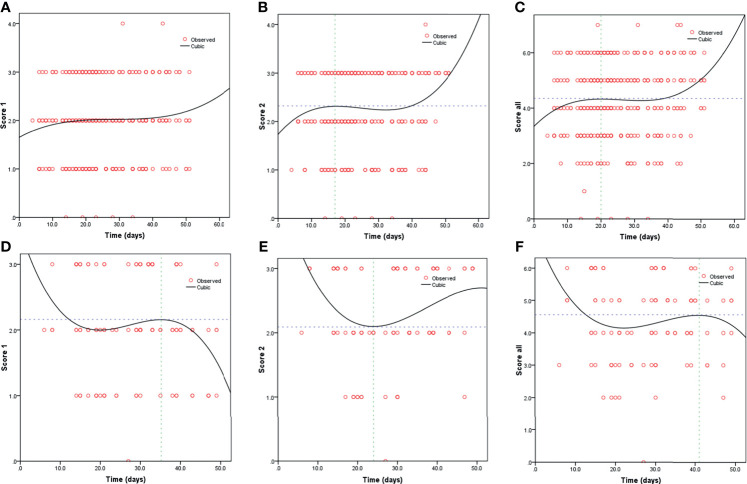
Fitting curves for patients with COVID-19 based on normal/elevated ALT levels and CT scores. Dynamic changes in patients with **(A)** CT score 1 and normal ALT; **(B)** CT score 2 and normal ALT; **(C)** total CT score and normal ALT; **(D)** CT score 1 and elevated ALT; **(E)** CT score 2 and elevated ALT; and **(F)** total CT score and elevated ALT.

Kaplan-Meier curves also demonstrated that patients with elevated levels of AST and AST/ALT were at a high risk of developing severe COVID-19. However, the ALT levels did not correlate with patient survival (**Figure 1**). Using the fitted curves, we observed rising trends in patients with varying levels of AST, ALT, and AST/ALT (**Figures 2A–C, E, F** and **3A–C, E**), excluding score 1 patients with elevated levels of AST (**Figure 2D**) and ALT (**Figure 3D**), patients with changes in the total score (**Figure 3F**), and patients with elevated levels of ALT.

## Discussion

In December 2019, we witnessed an outbreak of pneumonia (of unknown etiology) in Wuhan that rapidly spread through China and progressed into a pandemic. This disease was shown to affect multiple organs in patients, in addition to the lung, as research progressed (Huang et al., 2020; Li et al., 2020; Lu et al., 2020). In a pathology report, based on the minimal autopsies of three patients that died of COVID-19 in Chongqin, China, the authors indicated that the lungs of patients with COVID-19 manifested significant pathological lesions. Furthermore, moderate microvascular steatosis and mild lobular and portal lesions, were seen in liver biopsy specimens from COVID-19 cases (Yao et al., 2020). Thus, studies have discussed the involvement of liver function in determining the severity of COVID-19 (Xie et al., 2020; Zhao et al., 2020).

In this study, we studied the symptoms, laboratory test results after admission, treatment measures during hospitalization, and prognosis of 1,788 patients with COVID-19, including 15 fatal cases. Based on *t*-tests, the majority of biochemical laboratory results differed significantly between the patient groups. There was also a correlation between elevated AST levels and fever or fatigue; symptoms of respiratory failure influenced disease progression in patients with normal and elevated levels of AST. However, there were no significant differences between the patient groups in relation to other comorbidities. Furthermore, adjusted logistic and Cox regression analyses showed that levels of AST and AST/ALT, but not those of ALT, may serve as prognostic markers for increased disease severity and mortality in patients with COVID-19.

COVID-19 is caused by SARS-CoV-2, which similar to other coronaviruses (SARS-CoV-1 and MERS-CoV). Angiotensin-converting enzyme 2 (ACE2) is the receptor responsible for the internalization of severe acute respiratory syndrome coronavirus 2 (SARS-CoV-2). ACE2 is primarily expressed in the heart, kidney, and testes and expressed at basal levels in many other tissues (Hoffmann et al., 2020; Yan et al., 2020). A recent study has shown the binding between SARS-CoV-2 particles and ACE2 expressed by cholangiocytes. This triggers a systemic inflammatory response that leads to liver injury (Chai et al., 2020) and elevated levels of AST in patients with COVID-19. Furthermore, patients with severe disease exhibit a steep increase in the levels of AST, relative to other indicators of liver injury, thereby increasing the mortality of patients during hospitalization (Lei et al., 2020).

While the AST/ALT levels do not correlate with the histological parameters of disease severity in the liver, changes in this ratio indicate specificity in differentiation between cirrhotic and non-cirrhotic patients with chronic hepatitis C (Sheth et al., 1998). Studies have also shown that the development of primary biliary cirrhosis is associated with changes in AST/ALT (Nyblom et al., 2006). A study by Park et al., revealed that AST/ALT levels were valid predictors of cirrhosis, and that the AST/ALT ratio correlated with the histological grade of necroinflammatory activity and fibrosis (Park et al., 2000). In our study cohort, we found significant differences between patients with elevated AST/ALT levels in terms of severity/mortality. This suggests that patients with COVID-19 and elevated levels of AST/ALT need long-term follow-up, owing to their increased susceptibility to liver cirrhosis.

The fitted curves showed rising trends with increases in the duration of hospital stay for various patient groups. Thus, we speculated that, except for virus-induced liver injury during hospitalization, the use of drugs would significantly increase the risks of liver damage (Chau et al., 2004). Cai et al. showed that the use of lopinavir/ritonavir increased the odds ratio associated with liver injury by four-fold, suggesting the need to closely monitor patients who have been administered lopinavir/ritonavir (especially in patients with abnormal findings in liver function tests during admission) (Cai et al., 2020).

There are also limitations in our study cohort. We found a correlation between the levels of AST and AST/ALT and the prognosis of patients with COVID-19. However, contrary to the results of published studies, ALT levels were not significantly correlated with the prognosis of patients with COVID-19 (Lei et al., 2020). Thus, the involvement of the ALT level as a prognostic marker for patients with COVID-19 remains to be understood in detail. Fang et al. indicated that elevations in the levels of lactate dehydrogenase and gamma-glutamyl transpeptidase reflect bile duct injury; another thesis reported that the binding of ACE2 expressed by cholangiocytes to SARS-CoV-2 affects the liver (Lei et al., 2020; Xu et al., 2020). Thus, it is imperative to delineate the mechanism(s) employed by SARS-CoV-2 to alter liver function.

In conclusion, we have demonstrated that elevated levels of AST and AST/ALT may serve as markers for disease progression, poor prognosis, and high mortality. Moreover, the correlation between elevated levels of AST/ALT and poor long-term prognosis indicates the development of liver cirrhosis. Thus, clinicians should provide special attention to patients with COVID-19 with abnormal live function and encourage long-term follow-up after their recovery and discharge.

## Data Availability Statement

The raw data supporting the conclusions of this article will be made available by the authors, without undue reservation.

## Ethics Statement

This study was approved by the Research Ethics Commission of the Zhongnan Hospital, Wuhan University (approval number: 2020074), and patient consent was waived by the ethics committee as COVID19 is a rapidly evolving infectious disease.

## Author Contributions

All authors contributed to the design of the study and writing of the manuscript. QX and YG undertook the research. ZWL, QW, MLY, and YH performed the analyses and interpretation of data. ZML, DH, and JL wrote the main manuscript text and prepared figures. MW, LG, and XW revised the article critically for important intellectual content. All authors contributed to the article and approved the submitted version.

## Conflict of Interest

The authors declare that the research was conducted in the absence of any commercial or financial relationships that could be construed as a potential conflict of interest.

## Publisher’s Note

All claims expressed in this article are solely those of the authors and do not necessarily represent those of their affiliated organizations, or those of the publisher, the editors and the reviewers. Any product that may be evaluated in this article, or claim that may be made by its manufacturer, is not guaranteed or endorsed by the publisher.

## Abbreviations

COVID-19, coronavirus disease; AST, aspartate transaminase; ALT, alanine transaminase; CI, confidence interval; CT, computed tomography; SD, standard deviation; IQR, median and interquartile range; ACE2, angiotensin-converting enzyme 2; SARS-CoV-2, severe acute respiratory syndrome coronavirus 2.
